# Supplementary material for: A Smartphone-Enabled Continuous Flow Digital Droplet LAMP Platform for High Throughput and Inexpensive Quantitative Detection of Nucleic Acid Targets
Source: Sensors (Basel). 2023 Oct 8;23(19):8310. doi: 10.3390/s23198310 (PMC10575248; doi:10.3390/s23198310)
Supplement: Supplementary file 1 [file sensors-23-08310-s001.zip › sensors-2615377-supplementary.pdf]

Supporting Information

A Smartphone-Enabled Continuous Flow Digital Droplet LAMP Platform for High Throughput and Inexpensive Quantitative Detection of Nucleic Acid Targets

Elijah Ditchendorf[1], Isteaque Ahmed[1], Joseph Sepate[1] and Aashish Priye*[1,2]

Affiliations

[1] Department of Chemical and Environmental Engineering, University of Cincinnati, Cincinnati, OH 45221, USA

[2] Digital Futures, University of Cincinnati, OH 45221, USA

Table S1: LAMP Primer sequence for amplification of λ phage DNA

**Name**	Sequence (5`-->3`)	**Description**	**Total** length	**GC%**	**TM**
**FIP (F1c-F2)**	CAGCATCCCTTTCGGCATACCA-GGTGGCAAGGGTAATGAGG	FIP primer	41	56.1	69.5
**BIP (B1c-B2)**	GGAGGTTGAAGAACTGCGGCAG-TCGATGGCGTTCGTACTC	BIP primer	40	57.5	69.4
**F3**	GAATGCCCGTTCTGCGAG	F3 primer	18	61.1	56.7
**B3**	TTCAGTTCCTGTGCGTCG	B3 primer	18	55.6	55.2
**LF**	GGCGGCAGAGTCATAAAGCA	Loop forward primer	20	55	57.9
**LB**	GGCAGATCTCCAGCCAGGAACTA	Loop backward primer	23	56.5	60.4

All primer sequences from order from Integrated DNA Technologies (IDT)

Note S1: Image Analysis Code

```
% Initialize video capture settings
vidObj = videoinput('winvideo', 1, 'YUY2_1920x1080');
vidObj.FramesPerTrigger = 1;
vidObj.FrameGrabInterval = 1;
vidObj.FramesPerTrigger = Inf;
vidObj.FrameRate = '60';
triggerconfig(vidObj, 'manual');

% ISO and exposure
setCameraISO(vidObj, 400);
setCameraExposure(vidObj, 1/60);

% Start video capture
start(vidObj);

% Initialize variables
runTime = 30 * 60;  % 30 minutes in seconds
frameStack = [];
tic;

while toc < runTime
    % Capture a frame
    frame = getsnapshot(vidObj);

    % Stack frames for median computation
    frameStack = cat(4, frameStack, frame);

    % Separate into RGB channels
```

```
R = frame(:,:,1);
G = frame(:,:,2);
B = frame(:,:,3);

% Save RGB frames locally (simulating upload to MATLAB Online Drive)
save(['frame_', num2str(toc), '_R.mat'], 'R');
save(['frame_', num2str(toc), '_G.mat'], 'G');
save(['frame_', num2str(toc), '_B.mat'], 'B');

% Perform Gaussian noise reduction
kernelSize = [5, 5];
stdDev = 1.5;
R = imgaussfilt(R, stdDev, 'FilterSize', kernelSize);
G = imgaussfilt(G, stdDev, 'FilterSize', kernelSize);
B = imgaussfilt(B, stdDev, 'FilterSize', kernelSize);

% Compute median image for background subtraction
medianImage = median(frameStack, 4);

% Perform background subtraction
R = R - medianImage(:,:,1);
G = G - medianImage(:,:,2);
B = B - medianImage(:,:,3);

% Droplet detection using Hough Transform
[centers, radii] = findcircles(G, [10 300]);  % Adjust radii range as needed

% Calculate droplet sizes in micrometers
conversionFactor = 0.5;  % 0.5 micrometers per pixel
dropletSizes = radii * 2 * conversionFactor;
```

```
    % Morphological operations (erosion and dilation)
    se = strel('disk', 2);
    R = imerode(R, se);
    G = imerode(G, se);
    B = imerode(B, se);

    R = imdilate(R, se);
    G = imdilate(G, se);
    B = imdilate(B, se);

    % droplet fluorescence intensity measurements
red = R;
green = G;
blue = B;

[r,c]=size(green);

for i=1:r
    for j=1:c
        [X,Y,Z]=rgb_XYZ(red(i,j),green(i,j),blue(i,j));
        x(i,j) = X/(X + Y + Z);
        y(i,j) = Y/(X + Y + Z);
        Lum(i,j) = Y;

        % Map all points on 3D XYZ plot
        figure(2)
        switch (file_count)
            case 1
        plot(x(i,j),y(i,j),'*r','linewidth',1.5);
```

```
        break;
            case 2
                plot(x(i,j),y(i,j),'*g','linewidth',1.5);
                break;
            case 3
                plot(x(i,j),y(i,j),'*b','linewidth',1.5);
                break;
            case default
                plot(x(i,j),y(i,j),'*k','linewidth',1.5);
                break;
        end

    end
end

% Map average color point on 3D XYZ plot
figure(1)
pos_x =  mean(mean(x));
pos_y =  mean(mean(y));
Luminosity = mean(mean(Lum));
plot(pos_x,pos_y,'.k','linewidth',1.5);
text(pos_x,pos_y,label_string,'FontSize',font_size,'FontWeight','Bold','color',label_color); %plot the string

avg_red = mean(mean(red));
avg_green = mean(mean(green));
avg_blue = mean(mean(blue));

min_red = min(min(red));
min_green = min(min(green));
min_blue = min(min(blue));
```

```
max_red = max(max(red));
max_green = max(max(green));
max_blue = max(max(blue));

store_intensity(store_intensity_counter,1) = avg_red;
store_intensity(store_intensity_counter,2) = avg_green;
store_intensity(store_intensity_counter,3) = avg_blue;
store_intensity(store_intensity_counter,4) = pos_x;
store_intensity(store_intensity_counter,5) = pos_y;
store_intensity(store_intensity_counter,6) = Luminosity;
store_intensity_counter =  store_intensity_counter + 1;
clear red green blue x y X Y image_data;

    % Save processed data
    save(['processed_data_', num2str(toc), '.mat'], 'centers', 'radii', 'dropletSizes');
end

function [centers, radii] = custom_imfindcircles(I, radiiRange)
    % Convert image to grayscale if it's not already
    if size(I, 3) == 3
        I = rgb2gray(I);
    end

    % Initialize variables
    [rows, cols] = size(I);
    max_radius = radiiRange(2);
    min_radius = radiiRange(1);
    H = zeros(rows, cols, max_radius);
```

```
% Step 1: Edge detection using the Canny method
edges = edge(I, 'Canny');

% Step 2: Voting in Hough space
[edgeY, edgeX] = find(edges);

for r = min_radius:max_radius
   for edgeIndex = 1:length(edgeX)
      x = edgeX(edgeIndex);
      y = edgeY(edgeIndex);

      % Calculate the circle perimeter points for each edge point
      theta = 0:0.01:(2*pi);
      x0 = round(x - r * cos(theta));
      y0 = round(y - r * sin(theta));

      % Remove points that are outside the image boundary
      validIndices = (x0 > 0) & (x0 <= cols) & (y0 > 0) & (y0 <= rows);
      x0 = x0(validIndices);
      y0 = y0(validIndices);

      % Accumulate votes in Hough space
      for i = 1:length(x0)
         H(y0(i), x0(i), r) = H(y0(i), x0(i), r) + 1;
      end
   end
end

% Step 3: Identifying circle centers
H_max = imregionalmax(H);  % Find regional maxima in Hough space
```

```
    [centerY, centerX, radii] = ind2sub(size(H_max), find(H_max));
    centers = [centerX, centerY];
end


% Stop and clean up
stop(vidObj);
delete(vidObj);
clear vidObj;
```

Figure S1: Droplet generator chip

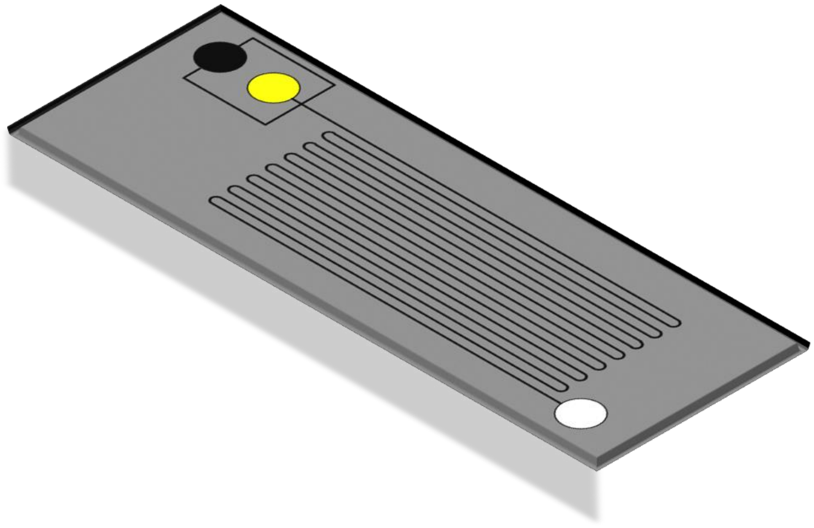

Figure S1: Droplet generator chip with two inlets (black inlet: continuous phase; yellow inlet: dispersed phase) and 15 serpentine channels and outline (white)

Figure S2: Smartphone optical setup

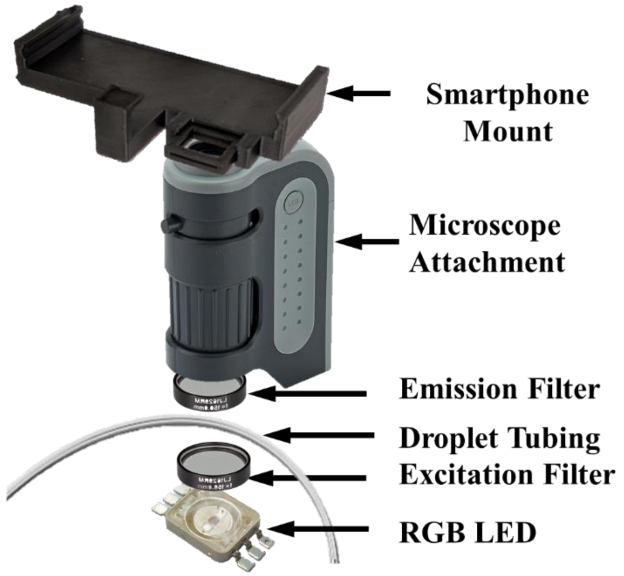

Figure S2: Smartphone optical setup: The smartphone attaches to the a mount which latches on to the 3D printed dark box housing the microscope attachment, emission filter, droplet tubing and excitation filter and RGB LED.
